# Alphavirus Replicon Particle Vaccine Breaks B Cell Tolerance and Rapidly Induces IgG to Murine Hematolymphoid Tumor Associated Antigens

**DOI:** 10.3389/fimmu.2022.865486

**Published:** 2022-05-24

**Authors:** Hsuan Su, Kazuhiro Imai, Wei Jia, Zhiguo Li, Rachel A. DiCioccio, Jonathan S. Serody, Jonathan C. Poe, Benny J. Chen, Phuong L. Doan, Stefanie Sarantopoulos

**Affiliations:** ^1^Department of Medicine, Division of Hematological Malignancies and Cellular Therapy, Duke University Medical Center, Durham, NC, United States; ^2^Department of Thoracic Surgery, Akita University Graduate School of Medicine, Akita, Japan; ^3^Biostatistics and Bioinformatics, Basic Science Department, Duke University Medical Center, Durham, NC, United States; ^4^Duke Cancer Institute, Duke University, Durham, NC, United States; ^5^Lineberger Comprehensive Cancer Center, University of North Carolina at Chapel Hill, Chapel Hill, NC, United States; ^6^Department of Medicine, University of North Carolina at Chapel Hill, Chapel Hill, NC, United States; ^7^Department of Microbiology and Immunology, University of North Carolina at Chapel Hill, Chapel Hill, NC, United States; ^8^Department of Immunology, School of Medicine, Duke University , Durham, NC, United States

**Keywords:** alphavirus replicon particle, hematolymphoid tumor, B cell tolerance, autoantigen, antitumor antibody, VRP, cancer vaccine

## Abstract

*De novo* immune responses to myeloid and other blood-borne tumors are notably limited and ineffective, making our ability to promote immune responses with vaccines a major challenge. While focus has been largely on cytotoxic cell-mediated tumor eradication, B-cells and the antibodies they produce also have roles in anti-tumor responses. Indeed, therapeutic antibody-mediated tumor cell killing is routinely employed in patients with hematolymphoid cancers, but whether endogenous antibody responses can be incited to blood-born tumors remains poorly studied. A major limitation of immunoglobulin therapies is that cell surface expression of tumor-associated antigen (TAA) targets is dynamic and varied, making promotion of polyclonal, endogenous B cell responses appealing. Since many TAAs are self-antigens, developing tumor vaccines that enable production of antibodies to non-polymorphic antigen targets remains a challenge. As B cell responses to RNA vaccines are known to occur, we employed the Viral Replicon Particles (VRP) which was constructed to encode mouse FLT3. The VRP-FLT3 vaccine provoked a rapid IgG B-cell response to this self-antigen in leukemia and lymphoma mouse models. In addition, IgGs to other TAAs were also produced. Our data suggest that vaccination with RNA viral particle vectors incites a loss of B-cell tolerance that enables production of anti-tumor antibodies. This proof of principle work provides impetus to employ such strategies that lead to a break in B-cell tolerance and enable production of broadly reactive anti-TAA antibodies as potential future therapeutic agents for patients with hematolymphoid cancers.

## Introduction

Monoclonal antibodies can be utilized to manipulate the immune response to tumors or to directly lead to tumor cell killing, especially when combined with chemotherapy by covalently linking the cytotoxic compounds to antibodies. Importantly, rapidly emerging chimeric antigen receptor (CAR) T cell and CAR NK cell therapies are also extending applications of B cell receptor (BCR) fragments that bind to tumor antigens (1–6). Thus, strategies that induce the active humoral immune response against tumor-associated antigens (TAAs) in the host have also received considerable attention because tumor cell killing is potentially mediated by antibody-dependent cellular cytotoxicity (ADCC) or cross-presentation of antigens to T cells. TAAs may emerge through mutations that lead to new epitopes in self-antigen (7–9). More commonly, TAAs are self-antigens that are either overexpressed or aberrantly expressed normal intracellular molecules. Because normal self-proteins are typically unrecognized by the host immune system unless there is a breach in immune tolerance, producing antibodies to TAA remains a major challenge, especially in tumors that circulate and potentially induce ongoing immune tolerance. If B cell responses to TAAs can be induced and harnessed, they can be used to develop potential therapeutic agents.

During the development of B cells, there is remarkable potential for BCR polyreactivity (10). Autoimmune disease can arise unless B cells with broadly reactive BCRs and the autoreactive antibody production are silenced by ongoing active mechanisms of tolerance that are operational in healthy individuals. In cancer patients, however, immune responses against self-antigens have been found (8). In addition, although many TAAs are self-antigens which are typically poorly immunogenic, antibodies against TAAs have been found in leukemia patients both before and after stem cell transplantation and in a variety of cancers (8, 9, 11–14). The tumor-specific antibodies found in cancer patients have been reported to be associated with better prognosis (9). Studies have also shown that B cells undergo clonal expansion, isotype switching, and affinity maturation within tumor-associated tertiary lymphoid structures (9). These findings further strengthen the role of the B cell response in antitumor immunity and suggest that the antitumor effects of B cells could be augmented through breaking the constraints of immune tolerance toward TAAs (15).

Promoting endogenous immune responses in the host that afford effective tumor eradication or enable development of antibody-based therapies is of potential high impact (16). Venezuelan equine encephalitis virus (VEE) is a positive, single-stranded RNA alphavirus. Packaged replicon particles derived from an attenuated strain of VEE (17, 18) have been used for the development of vaccines for infectious diseases and cancer (19, 20). These VEE replicon particles (VRP) are propagation-defective, but can infect lymphoid cells and have a tropism for dendritic cells (18, 21). They can only undergo one round of infection and are not able to replicate in the host. VRP encoding an inserted gene not only serve as efficient vectors that express the gene of interest in infected cells at high levels (17, 18, 22, 23), they have also been shown to activate both cellular and humoral immune responses against the encoded tumor antigens in murine models of breast tumor (24–27), melanoma (28, 29) and prostate cancer (30, 31). Clinical trials have shown that the VRP vaccines are well-tolerated and are safe to use in patients (32–34). Subsequent trials testing VRP-HER2 vaccines in breast cancer, and VRP-CEA vaccines in colon cancer patients are ongoing (35, 36). Without an inserted gene, VRP have been demonstrated to elicit strong adjuvant activity (21, 37–39). Given what is now known about B-cell activation and autoantibody production that is driven by nucleic acid receptor activation (40, 41), VRP utilization is considered an ideal approach that may provide signals through nucleic acid receptors and facilitate production of antibodies to the TAAs.

Eradication of melanoma utilizing a TAA-encoding VRP was in part mediated *via* ADCC in an Fc dependent manner (29). These responses do not appear to be impeded by host anti-vector immunity, and notably, repetitive vaccinations with VRP augment the anti-tumor responses (28, 31, 34). Given the efficacy of VRP in solid tumor models, we sought to evaluate the potential of VRP as an approach to vaccination against hematolymphoid malignancies. We chose the *fms*-like tyrosine kinase-3 (FLT3), a receptor tyrosine kinase that is involved in the proliferation and survival of hematopoietic stem cells and dendritic cells as a model TAA target in our studies. This is in part because studies have shown that FLT3 is highly expressed in most acute leukemias, and the internal tandem duplications (ITD) mutation of this gene that occurs in approximately 25% of AML cases, and increase FLT3 expression on malignant cells has been associated with an adverse prognosis (42, 43). Given the high rate of FLT3 mutations and prognostic impact, FLT3 has been recognized as an attractive target for immunotherapy.

Here we investigated the potency of VRP encoding FLT3 as a therapeutic vaccine in mouse hematolymphoid tumor models. We found that B cell responses against self-antigen FLT3 and other surface antigens expressed on tumor cells were induced in mice with established disease. We reveal the potential of using VRP vaccines in patients with leukemias or lymphomas, especially given that immune responses incited by single-antigen vectors appeared to lead to epitopes/antigen spreading. These findings also have promising intimations for the design of novel antibody-based immunotherapies for hematolymphoid malignancies. Our results warrant further study of VRP and other RNA-based viral particle vaccines in patients with leukemias and lymphomas.

## Material and Methods

### Animal and Cell Lines

B6.SJL and BALB/c mice were purchased from Taconic Biosciences Inc. (Germantown, NY) and Charles River (Wilmington, MA), respectively. 8- to 12-week-old female mice were used for all experiments. All experiments were performed under a protocol approved by Duke University Institutional Animal Care and Use Committee. C1498 and A20 were purchased from American Type Culture Collection (Manassas, VA). The pcDNA 3.1/myc-His vector (Invitrogen) harboring a gene encoding the extracellular and transmembrane domains and a short portion of the intracellular domain of mouse FLT3 (AA 1 to 584) was transfected into C1498 and A20 cells using Amaxa^®^ Cell Line Nucleofector^®^ Kit V (Lonza). The transfected cells were selected and maintained in RPMI medium containing 10% FBS, 10mM HEPES, 2 mM L-Glutamine, 1 mM sodium pyruvate, MEM non-essential amino acid, 0.055 mM 2-mercaptoethanol, and 400 μg/mL Geneticin™ (Gibco) at 37°C under 5% CO_2_. The FLT3 expression level on the transfected cells was determined by flow cytometry using fluorochrome-conjugated antibody against mouse FLT3/CD135 (Clone A2F10, BioLegend).

### Preparation of VRP

The extracellular-transmembrane domain of mouse FLT3 (AA 1 to 584) was cloned into the pVKE plasmid which was provided by Dr. Jonathan S. Serody (University of North Carolina at Chapel Hill, NC). Plasmid with the FLT3 transgene, pVKE-FLT3, or plasmid without the transgene, pVKE-mock, alone with other two helper plasmids encoding the VEE capsid and glycoprotein genes, respectively, were linearized by NotI (NEB) digestion, and T7 mMESSAGE mMACHINE^®^ Kit (Ambion) were used for *in vitro* transcription. Capped VRP transcripts were electroporated into Baby hamster kidney (BHK) cells (ATCC) under a condition of 1000V/100ohms/25uF/3 pulses using ECM^®^ 630 Exponential Decay Wave Electroporation System (BTX) for VRP packaging. The transfected cells were cultured with MEM-α medium (Gibco) containing 10% FBS, 2 mM L-Glutamine, 1 mM sodium pyruvate, pen/strep and 10% Tryptose Phosphate Broth (Sigma) at 37°C under 5% CO_2_ for 24-26 hours. Culture supernatants containing the VRP were harvested and clarified by centrifugation at 800 x g/4°C for 10 minutes. VRP were further concentrated by ultracentrifugation at 24,000 rpm/4°C for 3 hours through a 5 mL cushion of 20% (w/v) sucrose dissolved in PBS and were resuspended in PBS.

The titer (IU/mL) of VRP encoding the FLT3 gene (VRP-FLT3) or VRP lacking an inserted transgene (VRP-Ctrl) was determined by flow cytometry. BHK cell monolayers were infected with serial diluted VRP for 16-18 hours at 37°C under 5% CO_2_. Cells were harvested and counted with a hemacytometer. Cells were then fixed with Fixation/Permeabilization Solution Kit (BD Bioscience), followed by an incubation with 1:1000 diluted anti-VEE NSP mouse serum (Global Vaccine) at 4°C for 30 min. After being washed, cells were stained with 1:500 diluted Alexa Fluor^®^ 488-conjugated goat anti-mouse IgG antibodies (Clone Poly4053, BioLegend) at 4°C for 30 min. Stained cells were washed, suspended in FACS buffer, and analyzed by flow cytometry. The fraction of infected cells and the volume of VRP stock used for infection was plotted, and data points in the linear range were used for titer determination. The titer of VRP was calculated by the following equation:


$$\begin{array}{l} \left(\text{IU}/\text{mL}\right)=(\text{fraction of infected cell}\times \text{number of cells at time of infection})/(\text{volume of the viral stock used for infection}). \end{array}$$

### Tumor Challenge and VRP Vaccination

**Figure 1:**
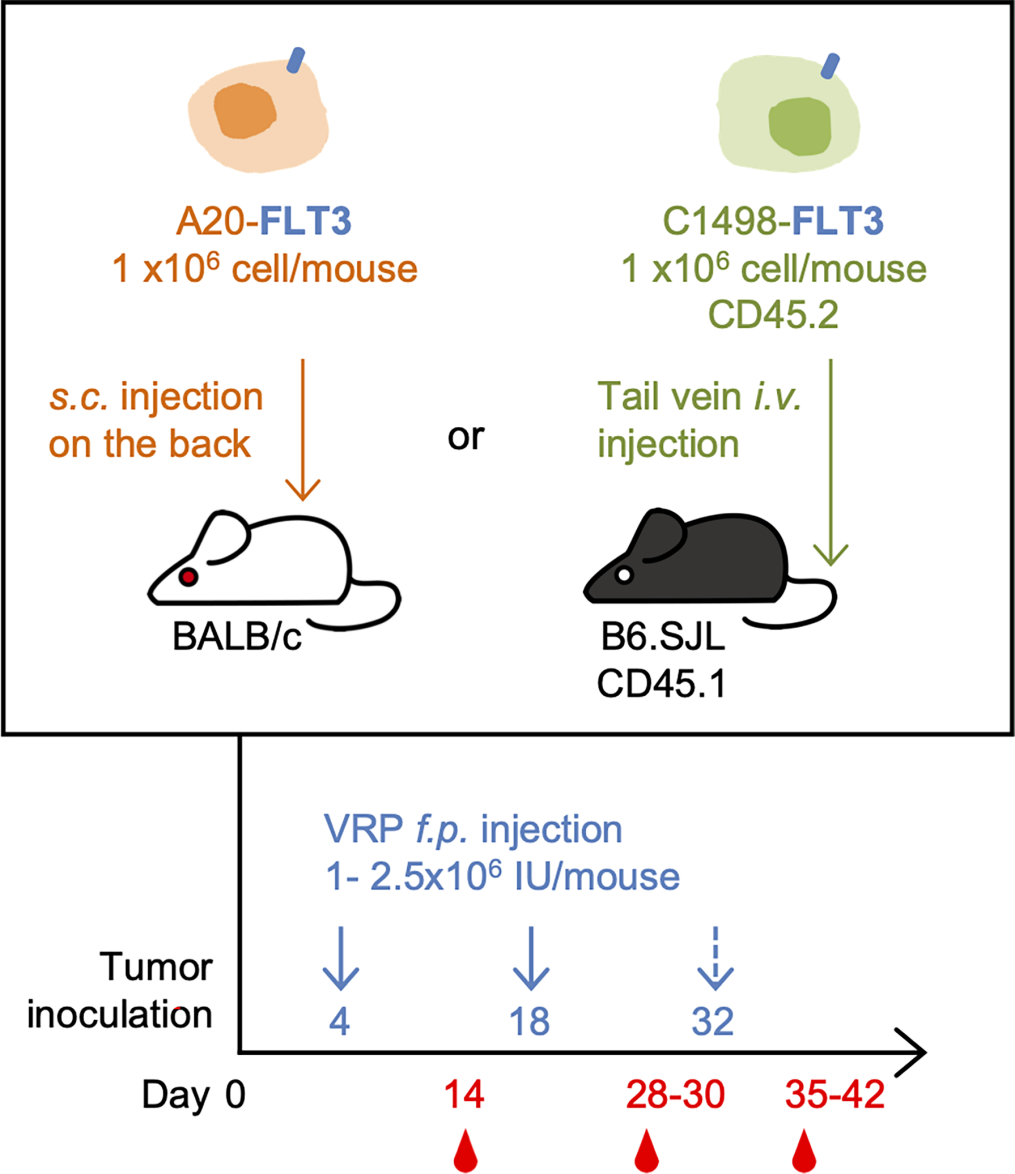
The mouse lymphoma model and leukemia model, and the VRP vaccination strategy. FLT3-expressing A20 tumor cells were inoculated subcutaneously on the back of BALB/c mice on day 0. Tumor-challenged mice were vaccinated on days 4 and 18 with 1 x 10^6^ IU of VRP-Ctrl or VRP-FLT3 on the footpad. Control mice received PBS (n= 5 per group). FLT3-expressing C1498 tumor cells were inoculated intravenously through tail vein of B6.SJL mice on day 0. Mice were vaccinated on day 4 and 18, and day 32 in one repeat of this model with 1-2.5 x 10^6^ IU of VRP-Ctrl or VRP-FLT3 on the footpad. The control mice received PBS (n= 5 or 10 per group). Blood samples were harvested on day 14, 28, and 35-42.

For C1498-FLT3 tumor model, CD45.1 congenic B6.SJL mice were inoculated with 1 x 10^6^ C1498-FLT3 cells intravenously *via* the lateral tail vein. For A20-FLT3 tumor model, BALB/c mice were subcutaneously inoculated with 1 x 10^6^ A20-FLT3 cells on the back. Tumor-bearing mice were vaccinated subcutaneously on the rear footpad with 1 - 2.5 x 10^6^ IU VRP in a volume of 20 uL PBS on days 4 and 18 post tumor inoculation. A third vaccine was given on day 32 post tumor inoculation in one repeat of the C1498-FLT3 tumor model. Mice in the vehicle control group received PBS only (**Figure 1**).

The expansion of C1498-FLT3 tumor in the blood of B6.SJL mice was detected using antibodies against mouse CD45.1 (Clone A20, BioLegend) and CD45.2 (Clone 104, BioLegend) by flow cytometry. Mice were monitored and were euthanized when hind limb paralysis or other signs of morbidity were observed to minimize the pain and discomfort. The growth of A20-FLT3 tumor was monitored by measuring two perpendicular diameters using a caliper. Tumor area was calculated according to the following formula: area (mm^2^) = length (mm) x width (mm). Mice were monitored and were euthanized when tumors reached an area of approximately 500 mm^2^, before reaching any further clinical signs and symptoms, in order to avoid unnecessary pain and discomfort. Maximum tumor size was always maintained within Duke IACUC guidelines.

### Flow Cytometric Analysis

For analysis of cell frequency and number in the blood, an equivalent volume of whole blood sample was drawn from the retro-orbital sinus of each mouse, and the blood was transferred immediately to tubes containing heparin (25 U/mL). After lysis of red blood cells with RBC Lysis Buffer (eBioscience), cells were then washed, and non-specific binding to Fc receptors was blocked with TruStain FcX (BioLegend) prior to the surface marker staining. Fluorochrome-conjugated antibodies against CD19 (Clone 6D5, BioLegend), CD4 (Clone GK1.5, BioLegend), CD8a (Clone 53-6.7, BioLegend), CD44 (Clone IM7, BioLegend) and CD62L (Clone MEL-14, BioLegend) were used for B or T cell population analysis. 7-AAD (BioLegend) was used for exclusion of dead cells, and a fixed volume of CountBright Absolute Counting Beads (ThermoFisher Scientific) were used for cell number calculation. Lymphocytes were gated based on the forward and side scatter parameters.

For detection of tumor-binding plasma IgG, 2.5 x 10^5^ tumor cells in each test were used. Cells were first stained with LIVE/DEAD™ Fixable Green Dead Cell Stain Kit (ThermoFisher Scientific). After washing, cells were blocked with TruStain FcX™ (BioLegend). Plasma samples from tumor-bearing mice were diluted to adjust the total IgG amount to 1 ug per test according to the result of total IgG ELISA, and were incubated with tumor cells at 4°C for 90 minutes. Cells were washed and followed by staining with 1:200 diluted fluorochrome-conjugated goat anti-mouse IgG antibodies (Clone Poly4053, BioLegend) and anti-mouse CD135 antibody (A2F10, BioLegend) at 4°C for 30 minutes. Stained cells were analyzed on a 3-laser BD FACSCanto II flow cytometer (BD), and the data were further analyzed using the FlowJo software (FlowJo, LLC).

### ELISA

Plasma samples were separated from blood by centrifugation at 600 x g/4°C for 10 minutes and were stored at -80°C until used. For plasma IgG determination, goat anti-mouse kappa (Southern Biotech, cat# 1050-01) and goat anti-mouse lambda (Southern Biotech, cat# 1060-01) antibodies were coated on the ELISA plate (Corning) at a total concentration of 2 μg/mL in coating buffer (pH 9.5, BioLegend) overnight at 4°C. Wells were washed with PBS containing 0.1% Tween-20 (PBST) and were blocked with PBST containing 0.5% (w/v) BSA (blocking buffer) at room temperature (RT) for 1 hour. Blocking buffer was also used as dilution buffer. After wells were washed, purified mouse IgG (BIO-RAD, cat# PMP01) was used as a standard in the assay and the plasma samples were serially diluted, and applied to the wells for a 2-hour incubation at RT. Wells were washed and were incubated with 1:5000 diluted HRP-conjugated goat anti-mouse IgG secondary antibody (Southern Biotech, cat# 1030-05) at RT for 1 hour. Wells were washed again, and TMB substrate (Thermo Fisher Scientific) was used according to the manufacturer’s instruction for color development. Reaction was stopped with 2M H_2_SO_4_ solution and the absorbance at 450nm was measured with the correction wavelength set at 570 nm. A standard curve with four-parameter logistic curve-fit was generated with GraphPad Prism software, and the IgG concentration of plasma samples was calculated according to the standard curve.

For the anti-FLT3 IgG, IgG1, IgG2b, IgG2c, and IgG3 ELISA, recombinant mouse FLT3 protein (rFLT3, Asn28 – Ser544, Sino Biological Inc.) diluted in coating buffer at a concentration of 2 ug/mL was coated on the ELISA plates overnight at 4°C. Wells were blocked with PBS containing 0.5% casein at RT for 1 hour. Plasma samples were serially diluted and applied to wells for a 2-hour incubation at RT. 1:5000 diluted HRP-conjugated goat anti-mouse IgG (Southern Biotech, cat# 1030-05), anti-mouse IgG1 (Southern Biotech, cat# 1070-05), anti-mouse IgG2b (Southern Biotech, cat# 1090-05), anti-mouse IgG2c (Southern Biotech, cat# 1079-05), or anti-mouse IgG3 (Southern Biotech, cat# 1100-05) was added to wells and incubated at RT for 1 hour. Between each incubation step, wells were washed with PBST. TMB substrate was used for color development as described above. The titration curves of anti-FLT3 plasma IgG were generated with four-parameter logistic curve-fit. Standard curves were generated from serial dilution of monoclonal mouse IgG1(Southern Biotech, cat# 0102-01), IgG2b (Southern Biotech, cat# 0104-01), IgG2c (Southern Biotech, cat# 0122-01), or IgG3 (Southern Biotech, cat# 0105-01). For the standard IgGs, goat anti-mouse kappa (Southern Biotech, cat# 1050-01) and anti-mouse lambda (Southern Biotech, cat# 1060-01) were coated as the capture antibodies. Absorbance values from the linear segment of each plasma dilution curve were used to interpolate the standard curve and calculate the IgG subclass concentration. (O.D value at 1:300 dilution of anti-FLT3 IgG1 and IgG3 ELISAs, and O.D. value at 1:2700 dilution of anti-FLT3 IgG2b and IgG2c ELISAs.)

For the tumor-binding IgG ELISA, membrane protein of C1498-mock cells was extracted using Mem-PER™ Plus Membrane Protein Extraction Kit (ThermoFisher Scientific), and was coated on plate at a concentration of 10 μg/mL in coating buffer (pH 9.5, BioLegend) overnight at 4°C. Wells were blocked with PBS containing 0.5% casein (BIO-RAD) at RT for 1 hour. Plasma samples were serially diluted and applied to the wells for a 2-hours incubation at RT. Wells were incubated with 1:5000 diluted HRP-conjugated goat anti-mouse IgG antibody (SouthernBiotech, cat# 1030-05) at RT for 1 hour. Wells were washed with PBST between each incubation step. TMB substrate was used for color development as described above. The titration curves were generated with four-parameter logistic curve-fit.

### Immunoblotting

For the Western blotting, rFLT3 protein (Sino Biological Inc.) was separated on NuPAGE Bis-Tris gel (ThermoFisher Scientific) under reducing conditions and transferred to nitrocellulose membranes using the iBlot system (ThermoFisher Scientific). Membranes were blocked with 1% casein protein in PBS (BIO-RAD) at RT for 1 hour, and were then cut into strips. Strips were incubated with rabbit anti-mouse FLT3 polyclonal IgG (LifeSpan BioSciences, Inc., cat# LS-C6858) or 1:250 diluted plasma samples overnight at 4°C. Strips were washed with PBST and were then probed with 1:15,000 diluted fluorochrome-conjugated antibody against rabbit IgG (H+L) (LI-COR, cat# 926-68073) or antibody against mouse IgG Fcγ fragment (Jackson ImmunoResearch, cat# 115-655-071) at RT for 1 hour. Strips were washed, rinsed with PBS and imaged by Odyssey infrared imaging system (LI-COR).

For dot blotting, 1 μL of rFLT3 protein diluted to a concentration of 50 ng/uL was spotted on the nitrocellulose membranes in duplicate and was dried on bench top for 6 min. Membranes were then blocked with 1% casein protein in PBS at RT for 1 hour, and were then cut into strips. Strips were incubated with 1:500 diluted plasma samples overnight at 4°C. Strips were washed with PBST and were then probed with fluorochrome-conjugated antibody against mouse IgG Fcγ fragment at RT for 1 hour. Strips were washed, rinsed with PBS and imaged by Odyssey infrared imaging system (LI-COR).

### Statistical Analysis

Significant differences in cell numbers or frequencies in blood, total IgG concentration, level of tumor antigen-specific IgG and MFI of tumor-binding IgG among experimental groups were determined by ordinary one-way ANOVA or Kruskal-Wallis test (non-parametric one-way ANOVA) as detailed in each figure legend. Significant differences in survival were determined by log-rank test. All statistical analyses were performed with GraphPad Prism software (Version 9.3.1). P-values less than 0.05 were considered significant.

## Result

### VRP-FLT3 Vaccination Induces Robust FLT3-Specific IgG Production in a Mouse Lymphoma Model

**Figure 2:**
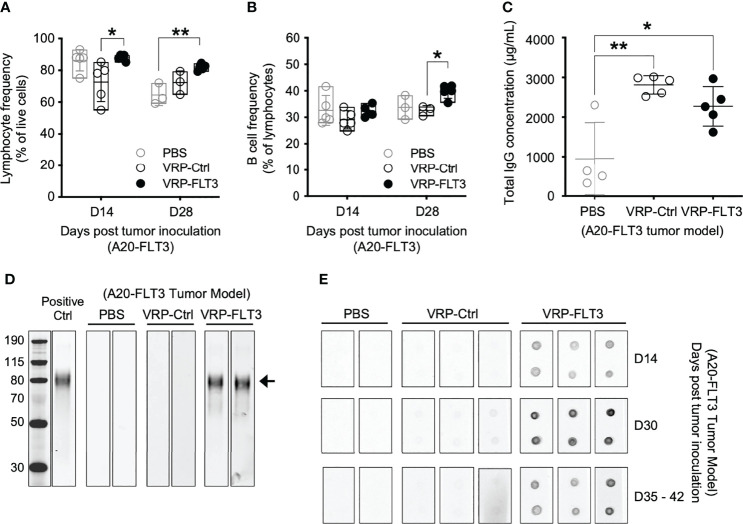
VRP-FLT3 vaccination elicits a FLT3-specific antibody response in a mouse lymphoma model. Mice challenged with A20-FLT3 tumor cells were vaccinated as described in **Figure 1**. **(A, B)** Blood samples harvested on days 14 and 28 were analyzed by flow cytometry for the frequencies of **(A)** lymphocytes and **(B)** CD19^+^ B cells. **(C)** Concentrations of total IgG in plasma on day 28 were determined by ELISA. Each symbol represents an individual mouse (n=5 per group). Bars represent the mean ± SD. Statistical significance was determined by one-way ANOVA, *P<0.05, **P<0.01. **(D)** Western blot of recombinant mouse FLT3 protein. Plasma collected on days 35-42 was 1:250 diluted and used for probing. Arrow indicates the FLT3 protein. Two representative samples per group are shown. Positive Ctrl represents FLT3 detection with commercial anti-FLT3 antibody. Panel at left represents MWM, with numbers indicating kDa for the bands shown. **(E)** Dot blot of recombinant mouse FLT3 protein. Protein was spotted in duplicate and probed with 1:500 diluted plasma collected on indicated time points. Two to three representative samples per group are shown. In both **(D, E)**, fluorochrome-conjugated anti-mouse IgG secondary antibody was used for signal development.

To investigate the ability of VRP vaccines to induce B-cell responses against a self-antigen, a mouse B cell lymphoma model was employed in our study. A20 cells were transfected with plasmid vectors harboring truncated mouse *Flt3* gene that contained only the extracellular, transmembrane, and a small part of the intracellular domain of *Flt3*. FLT3 antigen expression was confirmed by flow cytometry (**Supplementary Figure 1**) before tumor cells were subcutaneously inoculated on the back of BALB/c mice. FLT3-harboring VRP (VRP-FLT3) vs control VRP (VRP-Ctrl, vector alone) were administered by footpad injection as therapeutic vaccines on days 4 and 18 after tumor challenge. Blood was withdrawn on day 14 and 28 for analyzing the lymphocyte compartment of the tumor-bearing mice. With VRP-FLT3 vaccination, the frequency of lymphocytes was significantly increased (**Figure 2A**). Among the lymphocytes, an increase of B cell frequency on day 28 was observed only in VRP-FLT3 vaccinated mice (**Figure 2B**) but not VRP-Ctrl. By contrast, both VRP-FLT3 and VRP-Ctrl vaccinations induced a T cell response and resulted in a significant increase of activated CD44^+^ CD62L^-^ CD8^+^ T cell frequency (**Supplementary Figure 2A**).

To examine whether the significant increase in B cell number led to an anti-tumor B cell response after VRP-FLT3 vaccination in our mouse model, we measured antibody production in the tumor-bearing mice. Plasma samples collected on day 28 were analyzed for total IgG concentration, and we found that both VRP-FLT3 and VRP-Ctrl vaccinations significantly increased the total IgG level (**Figure 2C**). It has been shown that VRP possesses an adjuvant activity and promotes both cellular and humoral immune responses to co-delivered antigen (37, 44). The increase of total IgG in the VRP-Ctrl vaccinated mice could be a virus-related IgG response. To further evaluate the specificity of antibody to FLT3, we performed Western blot and dot bot with the rFLT3 protein. Plasma collected on days 35-42 was tested in Western blot. Only samples from VRP-FLT3 vaccinated mice contained IgG that recognized the recombinant FLT3 protein (**Figure 2D**). We then used a protein dot blot assay, where the rFLT3 protein remained in a native form, and we found a consistent result. Remarkably, by examining samples collected at early time points, we also found that the FLT3-specific IgG developed rapidly in tumor-bearing mice, as early as 10 days after the first VRP-FLT3 vaccination. Moreover, the titer of FLT3-specific IgG increased by a second vaccination and the response was sustained over time (**Figure 2E**). These findings suggest that the VRP-FLT3 vaccine induces a rapid IgG antibody response to the model TAA, a self-antigen, and the response can be boosted with repetitive vaccination.

### VRP-FLT3 Vaccination Alters the Composition of Circulating B Cells in a Mouse Lymphoma Model

**Figure 3:**
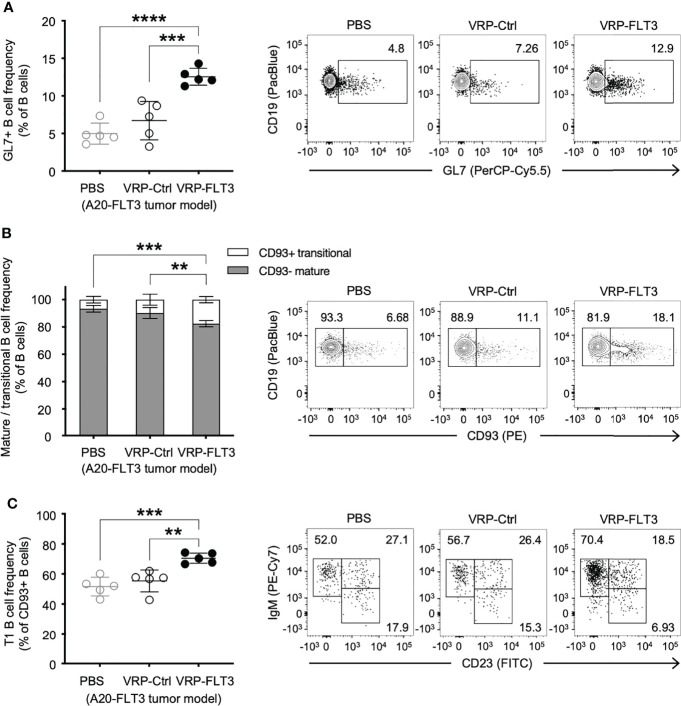
VRP-FLT3 vaccination alters the composition of circulating B cells in a mouse lymphoma model. Mice challenged with A20-FLT3 tumor cells were vaccinated as described in **Figure 1**. Blood samples harvested on days 14 were analyzed by flow cytometry for the frequency of **(A)** GL7^+^ B cells, **(B)** CD93^+^ mature B cells and CD93^–^ non-mature B cells, or **(C)** CD93^+^ IgM^+^ CD23^–^ T1 B cells (n=5 per group). A representative sample of each group is shown in contour plot on the right. Bars represent the mean ± SD. Statistical significance was determined by one-way ANOVA, **P<0.01, ***P<0.001, ****P<0.0001.

To investigate whether VRP vaccination affected certain B cells, we examined the composition of the peripheral B cell compartment using a flow cytometric read-out of serial blood from tumor-bearing mice that received control vs VRP-FLT3 vaccination. Having previously shown that the circulating GL7^+^ B cells were proportionally increased after alloantigen exposure when B-cell tolerance was lost in mice with chronic graft versus host disease, and production of host tissue-reacting antibodies was promoted (45, 46), we included GL7 in our B cell panel. In the current mouse lymphoma model, we found that the GL7^+^ B cell frequency was significantly increased in VRP-FLT3 vaccinated mice on day 14 after tumor inoculation **(** **Figure 3A****)**. Since the GL7^+^ B cell subset from secondary lymphoid organs has a higher capacity for antibody production compared to the GL7^-^ subset after reactivating with the same antigen used for immunization (47), this suggests that antigen-activated GL7^+^ B cells are incited by VRP vaccination.

In addition to the increase of GL7^+^ B cells, we found a significant elevation of CD93^+^ transitional-type B cells in the circulation after VRP-FLT3 vaccination by day 14 (**Figure 3B**), suggesting the B cell compartment was prone to autoreactivity. Moreover, within the CD93^+^ population, the frequency of IgM^+^CD23^–^ T1 transitional B cells was also significantly increased (**Figure 3C**). These data suggest breaking tolerance to self-antigen given that the overexpression of TLR7, an endosomal RNA sensor, was reported to promote the expansion of transitional-type B cells and autoantibody production (48). Taken together, data suggest that production of IgG to anti-self FLT3 induced by the VRP-FLT3 vaccine may reside in the expanded GL7^+^ or transitional B cell populations.

### VRP-FLT3 Vaccination Induces Robust FLT3-Specific IgG Production in Mouse Leukemia Model

**Figure 4:**
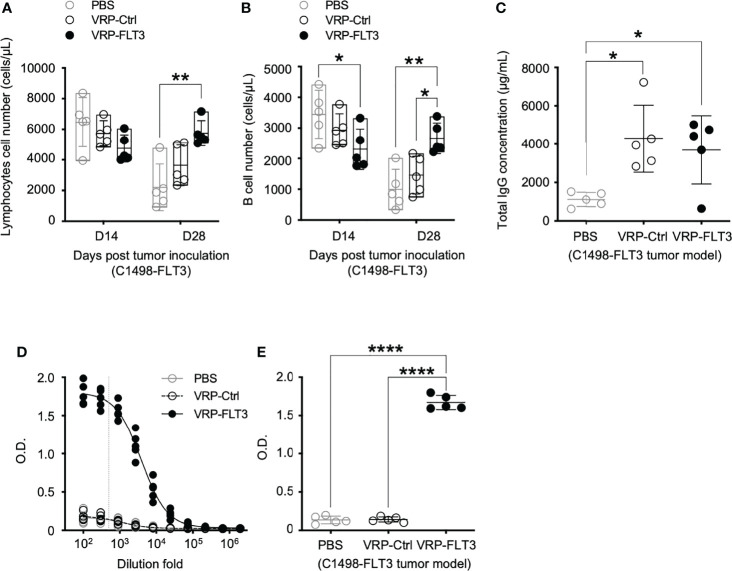
VRP-FLT3 vaccination elicits a FLT3-specific antibody response in a mouse leukemia model. Mice challenged with C1498-FLT3 tumor cells were vaccinated as described in **Figure 1**. **(A, B)** Blood samples harvested on days 14 and 28 were analyzed by flow cytometry for the numbers of **(A)** lymphocytes and **(B)** CD19^+^ B cells. **(C)** Concentrations of total IgG and **(D, E)** levels of FLT3-specific IgG in day 28 plasma samples were determined by ELISA. The titration curves **(D)** and O.D. values at 1:500 dilution **(E)**, indicated as the vertical dashed line in **(D)**, of anti-FLT3 IgG ELISA are shown. Each symbol represents an individual mouse (n=5 per group). Bars represent the mean ± SD. Statistical significance were determined by one-way ANOVA, *P<0.05, **P<0.01, ****P<0.0001. Data represents one of two independent experiments.

To investigate the potential use of VRP vaccines to break B cell tolerance in leukemia, a C1498 mouse leukemia model was employed. C1498 cells were transfected with plasmid vectors harboring truncated mouse *Flt3* gene that contained only the extracellular, transmembrane, and a small part of the intracellular domain of *Flt3*. FLT3 antigen expression on C1498-FLT3 cells was verified (**Supplementary Figure 1**) before the tumor cells were intravenously injected into B6.SJL mice. VRP-FLT3 or VRP-Ctrl vaccines were administered by footpad injection on days 4 and 18 after inoculation of C1498-FLT3 tumor cells. Peripheral lymphocyte compartments of these mice were then investigated for the immune responses elicited by VRP vaccines. With VRP-FLT3 vaccination, the number of lymphocytes in blood on day 28 was significantly increased (**Figure 4A**). Within the lymphocyte population, the B cell number in the peripheral blood of VRP-FLT3 vaccinated tumor-bearing mice was increased significantly on day 28 compared to that of the mice receiving PBS or VRP-Ctrl vaccines (**Figure 4B**). Besides the enrichment of B cells, the frequency of activated CD44^+^ CD62L^-^ CD4^+^ T cells and CD44^+^ CD62L^-^ CD8^+^ T cells was significantly higher in both VRP-FLT3 and VRP-Ctrl vaccination groups compared to the control mice that received PBS (**Supplementary Figure 2B**).

**Figure 5:**
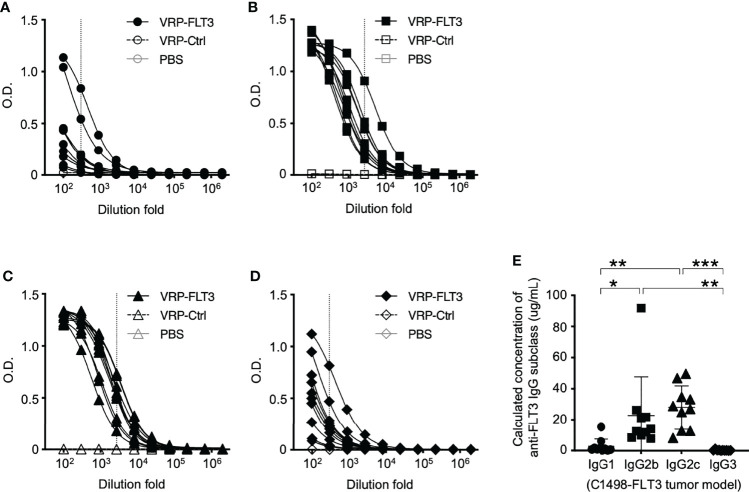
IgG2b and IgG2c are the predominant subclasses of FLT3-specific IgG induced by VRP-FLT3 vaccine. Antigen-specific IgG1 **(A)**, IgG2b **(B)**, IgG2c **(C)**, and IgG3 **(D)** responses were detected by ELISA using the recombinant FLT3 as the capture antigen. **(A–D)** Titration curves of samples was shown. One sample in PBS and VRP-Ctrl groups was tested, and ten samples in VRP-FLT3 groups were tested. **(E)** Levels of anti-FLT3 IgG subclasses were compared by calculating the equivalent IgG subclass concentration using O.D. values at 1:300 dilution of anti-FLT3 IgG1 and IgG3 ELIS as indicated as the vertical dash line in **(A)** and **(D)**, and O.D. values at 1:2700 dilution of anti-FLT3 IgG2b and IgG2c indicated as the vertical line in **(B, C)** ELISAs. Each symbol represents an individual mouse (n=10). Bars represent the mean ± SD. Statistical significance were determined by one-way ANOVA, *P<0.05, **P<0.01, ***P<0.001. Data represents one of two independent experiments.

While the B cell number in the blood was increased only in VRP-FLT3 vaccinated mice but not the VRP-Ctrl mice on day 28 after tumor inoculation, both VRP-FLT3 and VRP-Ctrl vaccines increased the total IgG level in tumor-bearing mice (**Figure 4C**). To investigate the production of antibody specific to FLT3 in tumor-bearing mice, we then employed a direct-binding ELISA using a recombinant mouse FLT3 (rFLT3) protein. A significant level of plasma IgG that recognized rFLT3 was detected in mice vaccinated with VRP-FLT3, while this was not observed in VRP-Ctrl vaccinated group (**Figures 4D, E****)**. Further investigation of the anti-FLT3 IgG induced by VRP-FLT3 vaccine showed that IgG2b and IgG2c, which are known to mediate ADCC, were the predominant subclasses among the FLT3-specific IgG (**Figure 5**). Thus, we demonstrated that VRP-FLT3 vaccine is capable of inducing a prompt IgG antibody response to FLT3 in both our lymphoma and leukemia mouse models. These data suggest that VRP-FLT3 can overcome the intrinsic B cell tolerance to self-antigen. In addition, the prevalence of IgG2b and IgG2c subclasses among FLT3-specific IgG hinted at a potential tumor-killing effect of the VRP-FLT3 vaccine through ADCC.

### Antibodies Against Tumor Membrane Antigens Are Induced by VRP-FLT3 Vaccination in Mouse Leukemia Model

**Figure 6:**
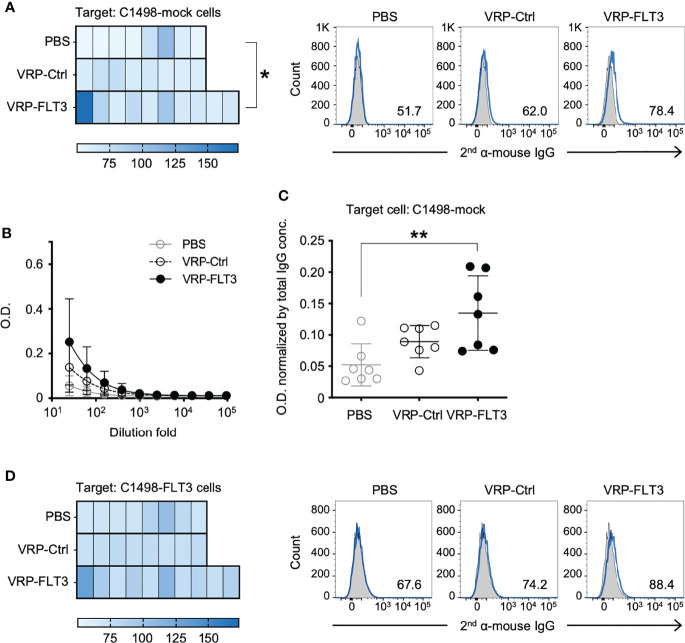
VRP-FLT3 vaccination induces broad reactivity of antibody to mouse leukemia cell membrane antigens. Mice challenged with C1498-FLT3 tumor cells were vaccinated as described in **Figure 1**. Plasma samples collected on days 35-42 were diluted and were incubated with **(A)** C1498-mock cells or **(D)** C1498-FLT3 at a level of 1 ug total IgG per reaction. The tumor cell-bound IgG was detected by a fluorophore-conjugated secondary anti-mouse IgG antibody using flow cytometry. Heatmaps of MFI of each sample are shown (n=8 or 10 per group). Statistical significance was determined by Kruskal-Wallis test (nonparametric) ANOVA, *P<0.05. A representative sample of each group is shown in Heatmaps on the right (2^nd^ antibody alone in shade grey and samples in blue line). **(B, C)** Plasma IgG bound to C1498-mock membrane extracts were determined by ELISA. Days 35-42 plasma samples were tested. **(B)** Titration curves (symbols represent the mean values in each group). **(C)** O.D. values obtained with 1:25 diluted plasma were normalized by the IgG concentration of each sample (O.D. / total IgG concentration). Each symbol represents an individual mouse (n=7 per group). Bars represent the mean ± SD. Statistical significance was determined by one-way ANOVA, **P<0.01.

The production of FLT3-specific IgG induced by VRP-FLT3 vaccine was confirmed by an ELISA using rFLT3. We then applied a flow cytometric approach to investigate the reactivity of plasma antibody to the tumor cells. Target cells used in this flow cytometric assays were the C1498-FLT3 cells or the non-FLT3 expressing C1498-mock cells. The FLT3 expression level of these target cells was confirmed (**Supplementary Figure 3**). Plasma samples collected on days 35-42 after tumor challenge were diluted according to the total IgG concentration to tailor the IgG amount in each test to 1 ug. Plasma IgG that reacted with tumor cells was detected by a fluorophore-conjugated secondary antibody. As epitope spreading or antigen spreading is observed in autoimmune diseases or cancer research (15, 49–52) and FLT3 is only one of numerous antigens expressed on tumor cells, we also thought to test whether VRP vaccines could elicit antibody responses against antigens other than FLT3. To test binding to other C1498 antigens, we performed the flow cytometric binding test C1498-mock cells as the targets (**Figure 6A**). Plasma samples from mice that received the VRP-FLT3 vaccination had higher levels of IgG bound to the C1498-mock tumor cells than plasma samples from PBS control mice. To verify this finding, we performed an ELISA with the membrane protein extracts of C1498-mock cells. Membrane protein extracts were coated on the plates, and plasma samples collected on days 35-42 from tumor-bearing mice were titrated and tested. We found that mice vaccinated with VRP-FLT3 developed a significantly higher level of IgG that reacted to tumor membrane proteins other than FLT3 compared to the PBS control mice (**Figures 6B, C****)**. These results indicate that VRP-FLT3 vaccinated mice had antibody responses toantigens other than the VRP encoded antigen, FLT3. When C1498-FLT3 cells was used as the target in the flow cytometric assay, plasma samples from VRP-FLT3 vaccinated mice tended to have overall higher reactivity to the C1498-FLT3 tumor cells than plasma samples from mice vaccinated with PBS or VRP-Ctrl (**Figure 6D**), although statistical significance was not reached in this assay, unlike clear demonstration of anti-FLT3 antibody production using purified protein in the FLT3 ELISA (**Figure 4E**). Together, data suggest that we found binding of IgG to antigen targets beyond the FLT3 model antigen.

### VRP-FLT3 Vaccination Attenuates Growth of Tumor in Both Mouse Lymphoma Model and Leukemia Model

**Figure 7:**
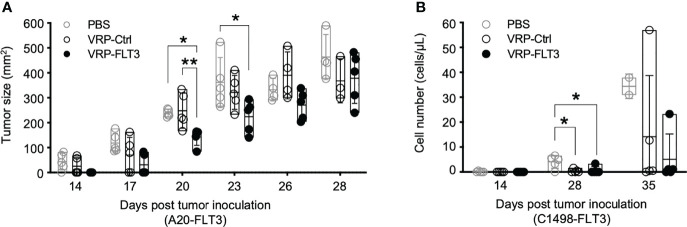
VRP vaccination attenuates the growth of FLT3-expressing A20 tumor cells and FLT3-expressing C1498 tumor cells. **(A)** Mice challenged with A20-FLT3 tumor cells were vaccinated as described in **Figure 1**. Tumor area of individual mice are depicted (n=5 per group). Lines represent the mean value of each groups. Statistical significance was determined by one-way ANOVA, *P<0.05, ^**^P<0.01. **(B)** Inoculation of FLT3-expressing C1498 tumor cells and the vaccination schedule are as described in **Figure 1**. C1498-FLT3 tumor cells in peripheral blood were determined by Flow cytometry. Each symbol represents an individual mouse (n=5 per group). Bars represent the mean ± SD. Statistical significance was determined by one-way ANOVA, *P<0.05.

Since we observed the induction of antibody responses to both FLT3 and other tumor membrane antigens, along with the activation of T cells, we assessed whether our VRP vaccine strategy targeted lymphoma and leukemia *in vivo*. Remarkably, after VRP vaccine we observed tumor size decrease in the A20-FLT3 tumor model. Tumor inoculated on the back of mice significantly decreased in mice vaccinated with VRP-FLT3 compared to the mice receiving PBS or VRP-Ctrl vaccines (**Figure 7A**). Likely related to the rapid proliferative capacity of this tumor, the VRP-FLT3 vaccine was unable to completely eliminate the tumor, and there was no survival benefit observed with either VRP-FLT3 or VRP-Ctrl vaccination (**Supplementary Figure 4A**). Attenuation of tumor growth was also observed in the C1498-FLT3 tumor model. We found an initial decrease in tumor cell number on day 28 after tumor challenge with both VRP-FLT3 and VRP-Ctrl vaccines. Importantly, tumor decrease was maintained at day 35 only in the VRP-FLT3 group although a statistical analysis was not possible since there were only 2 PBS-treated mice alive at day 35 (**Figure 7B**). Despite the rapid growth rate of C1498, there was also a significant improvement in survival of mice that received the VRP-FLT3 vaccine compared with the PBS control group (**Supplementary Figure 4B**). These data indicate that VRP vaccination attenuates the expansion of tumor cells *in vivo*.

## Discussion

Breaking tolerance to self-antigens is challenging but essential for vaccine approaches targeting TAAs that are recognized as self-antigens by the immune system in the cancer immunotherapy. Previous studies have demonstrated that VRP can induce specific antibodies to TAAs in studies of melanoma, breast cancer and colon cancer (24, 27–29, 34). While induction of T-cell responses with VRP has been reported in both animal and human studies of different solid tumors (27–31, 33–36), and this was also seen in our study, as the frequency of activated T cells increased in the blood of tumor-bearing mice with the VRP vaccinations, VRP is also known to elicit a potent humoral response as a vector system for delivering the antigen of interest or simply as an adjuvant co-administered with antigens (24, 26, 28, 29, 31, 34, 37, 39, 53). In our study, we now reveal the capacity of VRP to induce broadly reactive antibodies to self TAAs in two different hematolymphoid cancer mouse models. Here we clearly demonstrated that the VRP-FLT3 vaccination overcame the intrinsic self-tolerance mechanisms to induce a strong antibody response to FLT3 in the tumor-bearing mice in both of our leukemia and lymphoma models.

VRP encoding the TAA, FLT3, which is a self-antigen known to be overexpressed in acute leukemias, was given to mice bearing FLT3-expressing tumors. FLT3 was chosen as the target antigen in our study for inciting antitumor activity due to the high correlation between FLT3 and leukemia; however, there are many other TAAs that have yet to be discovered in blood cancers. Our study affirmed the reproducible induction of immune responses to a model self-antigen encoded by VRP, and we showed that the VRP system can be applied to other newly discovered tumor antigens or antigens with low immunogenicity for developing monoclonal antibodies. Antigen/epitope spreading induced by immunotherapy in cancer treatment has been reported (15, 50–52). In our study, in addition to the FLT3-specific antibody, we also found that vaccination of VRP-FLT3 can induce the production of antibodies reacting to the non-FLT3 expressing C1498-mock cells. The immunity to FLT3 elicited by VRP-FLT3 might serve as a trigger and raise a subsequent immune response against additional antigens on the tumor cells. Thus, here we demonstrate for the first time that VRP encoding FLT3 can promote a polyclonal B cell response to not only the encoded TAA but also other TAAs expressed on tumor cells. The observation of antigen/epitope spreading in our study hinted that the VRP system could potentially be used as a tool to explore unknown tumor antigens.

Both tumor cells types used in our lymphoma or leukemia models were aggressive and fast-growing. Thus, in our study, while the growth of tumors was attenuated by the VPR-FLT3 vaccination in both mouse models, complete tumor regression was not achieved. Intriguingly, we found prolonged survival in the leukemia mouse model, associated with a broadened B cell antibody response elicited by the VRP-FLT3 vaccine. In contrast to C1498, the more solid tumor-like A20-FLT3 lymphoma model may have very different tumor microenvironment from that of the C1498-FLT3 leukemia model. It has been shown that the anti-vector immunity to VRP did not hinder the efficacy of repetitive vaccination with VRP, and development of immunity to the encoded antigen increased as more VRP vaccinations were administered (28, 31, 33). Additional doses of VRP-FLT3 vaccine could potentially further boost and/or maintain the protection against the tumors in these mouse models. In solid tumor models, VRP encoding TAA can provide potent tumor protection in prophylactic settings in studies of melanoma, breast cancer, and prostate cancer (24, 25, 27–30, 54). However, the tumor protecting effect was relatively modest when tumors were established prior to the administration of VRP (29, 54). In some instances, failure in inhibiting the growth of preexisting tumors by VRP vaccine can occur despite the ability of VRP to induce some degree of TAA-specific immune responses (26, 30). One limiting factor in all of these studies, including ours, is the known low efficiency of viral packaging with the VRP system, whereby high titers of vector were not feasible. Technological advances in vaccine strategies with the advent of RNA vaccines will potentially overcome this limitation.

Consistent with our finding, VRP lacking a transgene has been shown to possess an adjuvant activity that promoted the immune response to co-delivered antigen (37, 44, 55). This could account for the increase in frequency of activated T cells in the periphery, and for the increase in total IgG level in serum of mice that received the VRP-Ctrl vaccine in our study. VRP-Ctrl vaccine, despite not encoding a transgene, was able to activate the immune system of tumor-bearing mice, yet it was unable to achieve TAA-specific responses. TAA-specific responses were only observed in tumor-bearing mice that received VRP-FLT3 vaccine, suggesting that a target antigen is needed to incite a desired immunity toward tumor cells. Others have shown that UV inactivation abrogated the adjuvant effect of VRP, indicating that viral RNA and/or the RNA replication plays an important role in triggering the immune responses (37). Several cellular sentinel sensors could be responsible for recognizing the viral replicative molecules (37, 56), and Toll-like receptor 7 (TLR7) is one of the candidates. TLR7 signaling in B cells has been related to activation of autoreactive B cells and induction of autoantibodies (40, 48, 57). Although a preferential infection of DCs and macrophages by VRP has been demonstrated, it has been shown that B cells can also be infected by VRP (21). The critical parameters that mediate the immune activity of VRP have not yet been fully elucidated and the mechanisms of B cell responses induced by VRP remain unclear, but our data suggest that a specific antigen response begets a broader one.

Combining other cancer treatments with VRP vaccine has been shown to yield a synergistic therapeutic activity (26, 54), indicating a possibility to shape the immune responses and enhance the antitumor efficacy of VRP vaccine. One previous study showed tumor reduction and prolonged tumor-free survival using the VRP-TRP2 vaccine in combination with either antagonist anti-CTLA-4 or agonist anti-GITR immunomodulatory mAb in a melanoma model (54).While checkpoint inhibitor is one of the options for developing new immune therapies, another future strategy may be to administer VRP vaccines in the context of hematopoietic stem cell transplantation (HCT), which still is an important treatment for hematolymphoid malignancies.

Combining autologous HCT (auto-HCT) with a VRP-TAA vaccine strategy to break immune tolerance to tumor antigens warrants further study. Patients with refractory blood cancers are often referred to allogeneic HCT (allo-HCT). Tumor-reacting antibodies were found in patients who underwent allogeneic HCT (14), and an antibody-mediated autoimmune-like syndrome, chronic graft versus host disease (cGVHD) developed after allo-HCT (58, 59), is known to be associated with decreased cancer relapse (60). However, cGVHD is also the main cause of morbidity and mortality in this type of HCT. In contrast, patients who receive auto-HCT do not develop cGVHD, but often encounter tumor relapse. Nevertheless, autologous HCT can be a strategy for delivery of high-dose chemotherapy to patients. It is known that myeloablative treatment before HCT induces a lymphopenia condition in which the B cell compartment reconstitutes in a setting of a high B-cell activating factor (BAFF)-to-B cell ratio and altered B cell homeostasis (61, 62). Our study demonstrated the possibility of provoking immunity to cancers. Applying the VRP-TAA vaccine, which has proven to be an effective treatment to alter self-tolerance, at the right time point after auto-HCT may enable the rescue of those potentially tumor-reactive B cells and augment more efficient anti-tumor immunity without the immune toxicity found after allo-HCT. Our results suggest that future studies addressing how VRP vaccine or other RNA vaccines that promote anti-TAA responses by B cells can be employed as a strategy to develop antibody therapies.

## Data Availability Statement

The original contributions presented in the study are included in the article/**Supplementary Material**. Further inquiries can be directed to the corresponding author.

## Ethics Statement

The animal study was reviewed and approved by Institutional Animal Care and Use Committee of Duke University.

## Author Contributions

SS conceptualized and supervised the study. SS, WJ, JP, and KI designed the experiments. WJ, KI, HS, and RD performed experiments and acquired data. KI and HS analyzed and interpreted results. ZL performed the statistical analysis. JS provided technical and material support. HS and SS wrote the manuscript. JP, BC and PD provided scientific advice and reviewed the paper. All authors contributed to the article and approved the submitted version.

## Funding

This work was funded by the Department of Defense, Congressionally Mediated Research Program W81XWH1-8-1-0239 (CA171068).

## Conflict of Interest

The authors declare that the research was conducted in the absence of any commercial or financial relationships that could be construed as a potential conflict of interest.

## Publisher’s Note

All claims expressed in this article are solely those of the authors and do not necessarily represent those of their affiliated organizations, or those of the publisher, the editors and the reviewers. Any product that may be evaluated in this article, or claim that may be made by its manufacturer, is not guaranteed or endorsed by the publisher.
