# Supplementary material for: Alphavirus Replicon Particle Vaccine Breaks B Cell Tolerance and Rapidly Induces IgG to Murine Hematolymphoid Tumor Associated Antigens
Source: Front Immunol. 2022 May 24;13:865486. doi: 10.3389/fimmu.2022.865486 (PMC9171395; doi:10.3389/fimmu.2022.865486)
Supplement: Supplementary file 1 [file DataSheet_1.docx]

Supplementary Material

## Supplementary Figures

**Supplementary Figure 1. FLT3 expression level on tumor cells.**

A20 and C1498 tumor cell lines were transfected with mouse FLT3. The FLT3 expression levels were determined by flow cytometry. Grey shade represents the isotype control. Solid line represents the sample.

**Supplementary Figure 2. VRP vaccine induces T cell response in tumor-bearing mice.**

Tumor inoculation and VRP vaccination schedule are as described in Fig 1. Blood samples harvested on days 14 and 28 were analyzed by flow cytometry for the frequencies of activated CD4+ and CD8+ T cells (CD44+ CD62L–). (A) A20-FLT3 tumor model (n=5 per group). (B) C1498-FLT3 tumor model (n=10 per group). Each symbol represents an individual mouse. Bars represent the mean ± SD. Statistical significance were determined by one-way ANOVA, *P<0.05, **P<0.01, ****P<0.0001.

**Supplementary Figure 3. FLT3 expression level of tumor cells that were used as the target cells in the flow cytometric binding assay.**

FLT3-expressing C1498 cells (left) or the non-FLT3 expressing C1498-mock cells (right) were incubated with diluted plasma collected from C1498-FLT3 tumor bearing mice. Tumor cell-reacting plasma IgG were detected by a secondary antibody. Grey shade represents the isotype control. Solid line represents the sample.

**Supplementary Figure 4. VRP vaccine improves the survival of tumor-bearing mice in the leukemia model but did not show a significant survival benefit in the lymphoma model.**

Tumor inoculation and VRP vaccination schedule are as described in Fig 1. Kaplan-Meier survival curves of **(A)** A20-FLT3 tumor inoculated mice (n=5 per group) and **(B)** C1498-FLT3 tumor inoculated mice (n=5 per group). Arrows indicate the days of VRP vaccination. Statistical significance was determined by Log-rank (Mantel-Cox) test, **P<0.01. For the C1498-FLT3 tumor model, the best of two independent experiments is depicted. The repeat experiment of the C1498-FLT3 tumor model included 10 mice per group and was ended on day 40 after tumor inoculation.
